# Development of a calorie-based weight prediction equation for Anorexia nervosa: a case report

**DOI:** 10.1186/s40337-025-01520-7

**Published:** 2026-01-09

**Authors:** Riito Fujimoto, Naohiro Arai, Tomoyuki Imai, Yuta Oshima, Minoru Takebayashi, Shuken Boku, Noboru Fujise

**Affiliations:** 1 NHO Kumamoto Medical Center, Kumamoto, Japan; 2 Department of Neuropsychiatry, School of Medicine, Keio University, 35 Shinanomachi, Shinjuku-ku, 160-8582 Tokyo Japan; 3 Department of Neuropsychiatry, Faculty of Medicine, Kumamoto University, 1-1-1 Honjo, Kumamoto, 860-8556 Japan; 4 Kamisuwa Hospital, Suwa, Japan; 5 Department of Psychiatry, Shonan Kamakura General Hospital, Kamakura, Japan; 6 Tamana Hospital, Kumamoto Tamana, Japan; 7 Department of Psychiatry and Neuroscience, Center for Metabolic Regulation of Healthy Aging, Faculty of Life Sciences, Kumamoto University, Kumamoto, Japan; 8 Department of Psychiatry, Niigata University Graduate School of Medical and Dental Sciences, Niigata, Japan; 9 Mental wellness Support Center, Kumamoto University Hospital, 1-1-1 Honjo, Kumamoto, 860-8556 Japan

**Keywords:** Anorexia nervosa, Weight gain, Body weight prediction model, Case report

## Abstract

**Background:**

Anorexia nervosa (AN) often requires nutritional rehabilitation including nasogastric feeding. However, achieving shared treatment goals between clinicians, patients, and families can be challenging. Weight prediction can be a valuable tool in this process; however, conventional approaches rely largely on clinical experience and lack precision. We therefore developed a simplified calorie-based weight prediction model grounded in the revised Harris–Benedict equation.

**Case presentation:**

This case report describes the clinical application of this model in a woman in her twenties with AN. By visualizing predicted weight trajectories under different caloric intake strategies, the model facilitated consensus building between the patient, her family, and the treatment team, leading to the acceptance of nasogastric feeding and structured behavioral therapy.

**Conclusions:**

The present case may suggest the potential utility of a calorie-based weight prediction model as a dynamic, noninvasive, and quantitative tool for inpatient management of AN. While the approach could facilitate understanding and agreement regarding treatment for AN, further evaluation in larger cohorts is required to assess accuracy and clinical impact.

**Supplementary Information:**

The online version contains supplementary material available at 10.1186/s40337-025-01520-7.

## Background

Anorexia nervosa (AN) is an eating disorder characterized by significantly low body weight, body image disturbance, and an intense fear of weight gain [1]. AN is more prevalent among young women and is associated with a high mortality rate, often due to medical complications [2, 3]. Although psychological therapy is an important component of treatment, as described later, nutritional rehabilitation needs to be provided concurrently or even prior to psychological intervention when especially severe emaciation or medical instability is present. However, its implementation often encounters challenges, including ambivalence toward weight restoration, fear of rapid weight gain, and disagreement between clinicians, patients, and families regarding the need for nasogastric feeding or structured behavioral therapy [4, 5]. These difficulties may delay the initiation of appropriate nutritional support and compromise medical stability. Although some individuals with AN require non-oral nutritional supplementation, they sometimes resist interventions such as nasogastric feeding. Patients who require compulsory nutrition have been noted to lack awareness of the seriousness of the illness and its associated medical risks, further complicating clinical decision-making [6].

There is substantial evidence supporting the effectiveness of psychological therapies in the treatment of adult AN, such as Enhanced Cognitive Behavior Therapy, Specialist Supportive Clinical Management, and the Maudsley Model of Anorexia Nervosa Treatment for Adults [7]. Although pharmacotherapy and neuromodulation have also been reported as potentially beneficial, the supporting evidence remains limited [4]. In addition to these treatments, nutritional rehabilitation is recognized as a component for initiating care in typical AN [4, 5]. Stepwise enteral or parenteral nutrition is often required [8–10], and weight restoration has been shown to be a key predictor of treatment outcome [11, 12]. However, initiation of treatment is frequently hindered by poor insight, resistance from the patient [13], and lack of understanding from the family [14]. Patients with AN may engage in compensatory behaviors such as purging, which can lead to serious systemic organ complications [15], highlighting the need for supplementary tools that can promote patients’ and families’ agreement and understanding of nutritional therapy.

The promising supplementary tool for nutritional therapy should be capable of presenting multiple scenarios in a transparent manner, based on basic data obtained in routine clinical practice, and in a form that is easily understood by patients and their families. However, conventional approaches have been dependent on estimation of body weight based on clinical experience [16], making it difficult to adequately convey the implications of different nutritional strategies for individual patients. The Harris-Benedict equation has sometimes been applied as a single-point calculation to estimate caloric intake and used as an adjunct to nutritional therapy [17]. However, this approach is limited to estimating energy expenditure at one point in time and cannot sequentially or dynamically predict changes in body weight. Instead of repeatedly applying a simple equation in isolation, a more objective, calorie-based assessment of body weight may be achieved by developing a mathematical model based on the Harris-Benedict equation and time-dependent changes in body weight. Establishing such a mathematical model allows for the integration of multiple clinical parameters, visualization of predicted outcomes, and explicit indication of the associated uncertainty. These characteristics of the models may facilitate the sharing of appropriate treatment goals and enhance consensus building among patients, families, and healthcare providers. However, to date, no reports have described the application of a calorie-based weight prediction model specifically adapted for patients with AN.

In the present report, we propose a simplified weight prediction model based on the revised Harris-Benedict equation and present a case in which this model was applied to facilitate treatment initiation, support shared decision making between the patient, family, and clinicians, and enable behavioral evaluation in a patient with AN, demonstrating its clinical utility.

## Methods

Written informed consent for publication of this case report was obtained from the patient. All personally identifiable information not essential for the case description was excluded to maintain confidentiality.

**Fig. 1:**
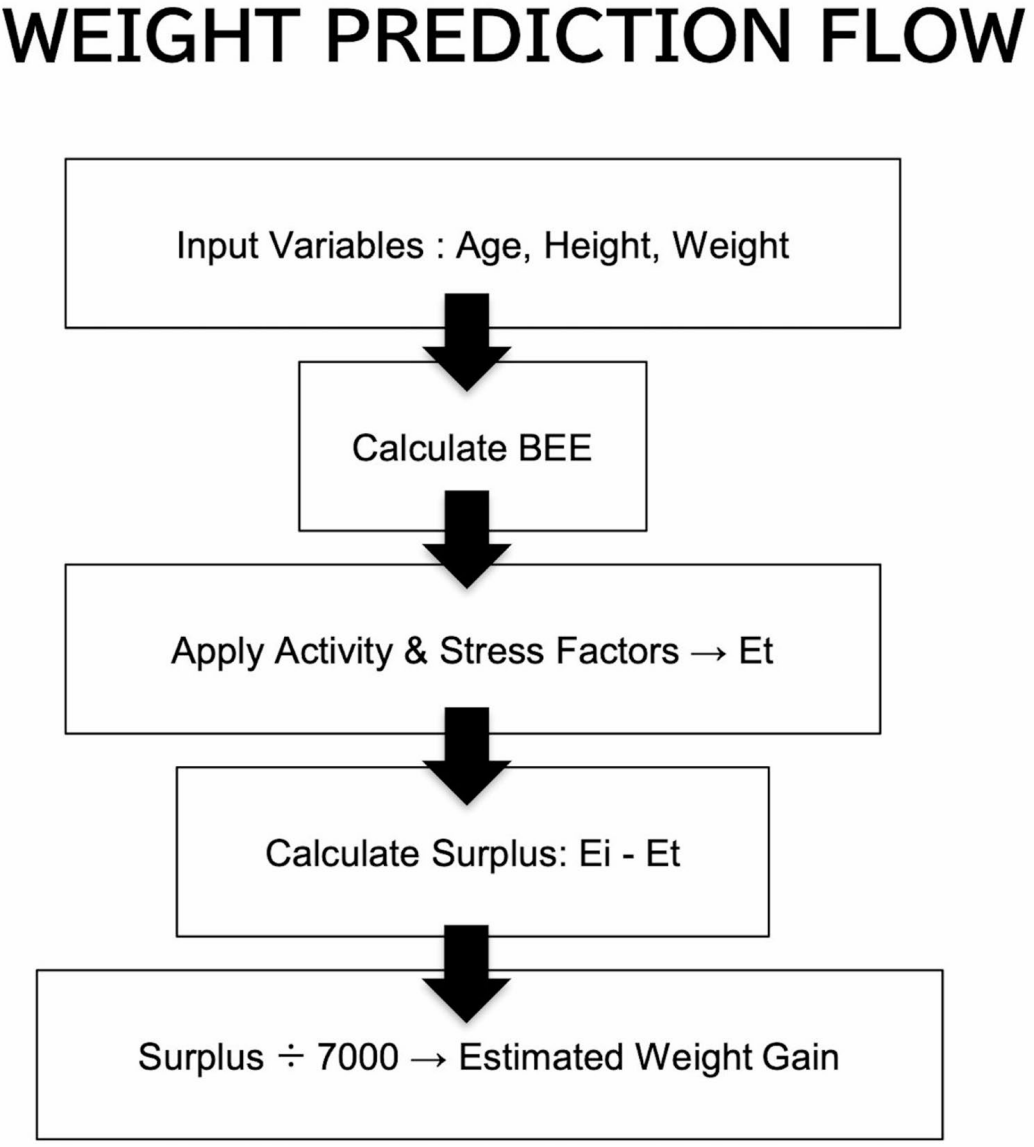
Calculation flow for the weight prediction equation. The flowchart illustrates the procedure for predicting daily body weight change. First, Basal Energy Expenditure (BEE) is calculated using the revised Harris-Benedict equation, incorporating current body weight, height, age, and sex. BEE is then multiplied by an activity factor and stress factor to estimate Total Energy Expenditure (Et). The difference between daily caloric intake (Ei) and Et is divided by 7000 kcal, the approximate energy equivalent of 1 kg of body weight, to estimate daily weight change. This recursive calculation is repeated over the desired number of days to project future body weight. *BEE* Basal Energy Expenditure, *Ei* caloric intake *Et* Energy Expenditure

We created a simplified weight prediction model based on the revised Harris-Benedict equation with the variables of age, height, and weight from the patient with AN. The calculation procedure for the model is illustrated in Fig. 1. Further computational details are provided in the Supplementary information.

## Case presentation

### Patient information

The patient was a woman in her twenties who was admitted to the hospital with liver dysfunction associated with extreme underweight. She lived with both of her parents and had no history of selective eating during childhood. There were no other medical or psychiatric comorbidities. She had a seven-year history of an eating disorder and had experienced multiple hospitalizations and discharges.

### Clinical course

At age 15, during her third year of junior high school, she joined the track and field club. After noticing performance improvements associated with weight loss, she became increasingly preoccupied with dieting. In her first year of high school, concerned by her significant weight loss, her parents brought her to a local psychosomatic medicine clinic, where she was diagnosed with AN. Subsequently, she developed marked elevations in alanine aminotransferase, exceeding 2,000 U/L, and was admitted to a general hospital for treatment.

Following discharge, she continued outpatient care at a local psychiatric clinic. However, her weight continued to decline, and at age 20 she was referred to our hospital. At the initial visit, her body mass index (BMI) was approximately 10, with laboratory findings showing elevated liver enzymes and leukopenia. She was admitted to our department for inpatient treatment. Through oral intake and psychotherapy, her weight increased to around 24 kg (BMI: 11.2), but then plateaued. She was discharged at a body weight of 24.6 kg (BMI: 11.5) at the strong request of the patient and her family.

Although she continued outpatient follow-up every two weeks, her weight did not improve. Three months later, she developed diarrhea after consuming raw horse meat and was readmitted due to low body weight and liver dysfunction.

### Assessment at re-admission

Upon readmission, her height was 146.2 cm, her weight was 21.1 kg, and her BMI was 9.9. Laboratory tests revealed liver dysfunction, leukopenia, and dehydration. Electrolytes, including phosphorus, were within normal limits, and infectious causes such as viral hepatitis were ruled out. Fluid replacement led to a rapid normalization of her laboratory abnormalities.

### Intervention during the initial treatment

At the request of the patient and her family, a one-month trial of oral feeding alone was conducted. During the trial, her daily caloric consumption was approximately 1,200 kcal/day, which was insufficient to achieve weight gain compared with the caloric levels provided through nasogastric feeding (approximately 2,000 kcal/day). Both the patient and her family were strongly committed to oral feeding and doubtful about the effectiveness of nasogastric feeding.

### Intervention during the mid-phase of treatment (Fig. 2)

**Fig. 2:**
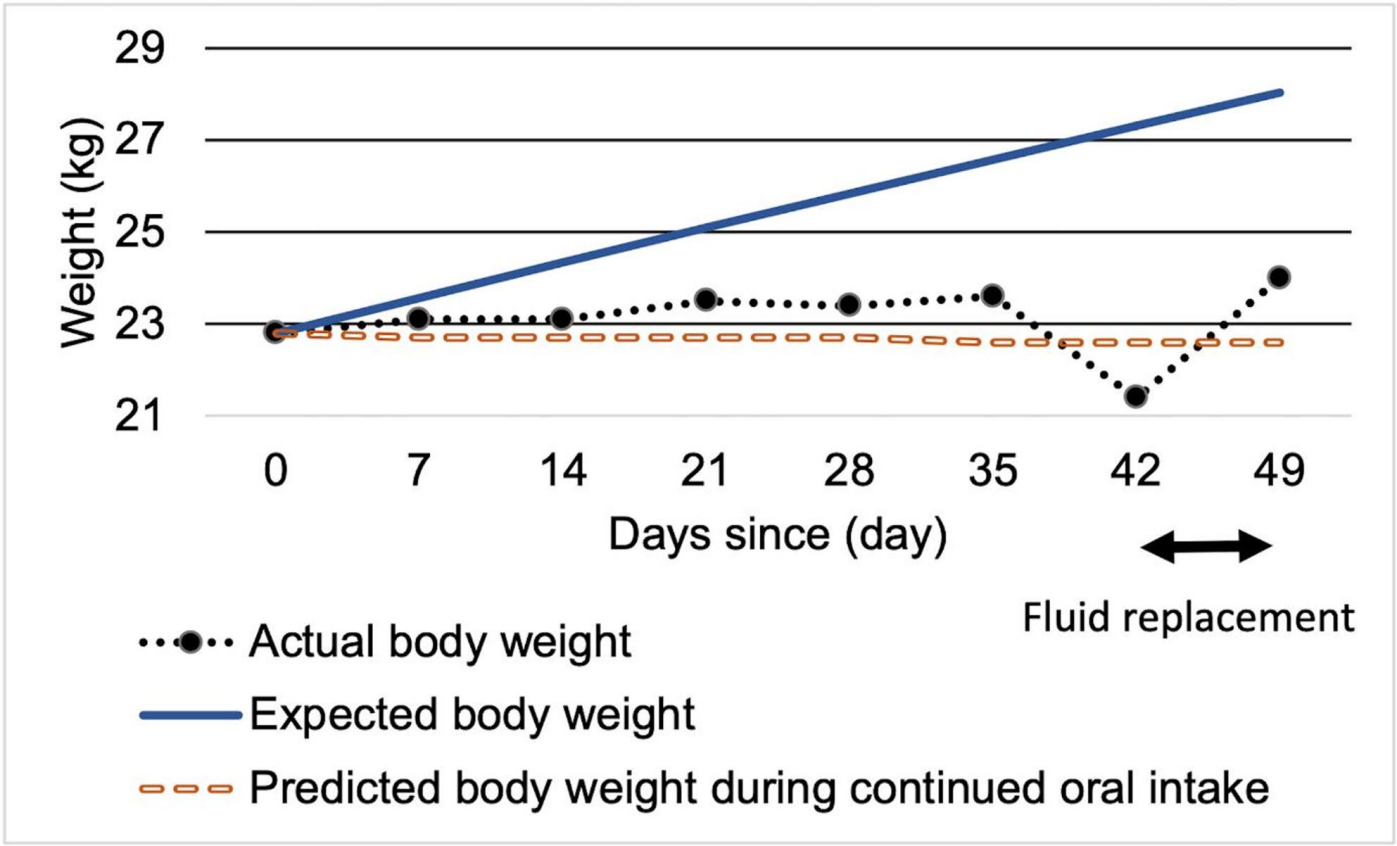
Weight trajectory before starting behavioral therapy. Body weight changes before the initiation of behavioral therapy. Solid line: predicted weight (2000 kcal/day); dotted line: actual weight; dashed line: predicted weight with oral intake only (1200 kcal/day). Intravenous fluids were administered on days 42–49 due to dehydration

To address concerns about oral versus nasogastric feeding, we developed a weight prediction model based on the revised Harris-Benedict equation and visually presented the predicted body weights corresponding to different caloric intake levels. By comparing projected weights under oral feeding alone versus oral feeding combined with enteral nutrition (Fig. 2), the patient and her family came to understand the limitations of oral feeding and the necessity of enteral nutrition via nasogastric tube. With the patient’s consent, nasogastric feeding was initiated (Fig. 2, Day 0).

Despite weekly weight monitoring, the expected weight gain was not achieved (Fig. 2, Day 35), and liver dysfunction re-emerged. The discrepancy between the predicted and actual weights suggested her compensatory behaviors such as excessive physical activity, food disposal, or self-induced vomiting. The patient’s excessive water intake was identified (Fig. 2, Day 35). Following the introduction of strict fluid intake and output monitoring, it became evident that her actual weight was even lower than previously measured (Fig. 2, Day 42).

By reapplying the weight prediction model, we demonstrated the impact of compensatory behaviors, enabling her and her family to clearly recognize the necessity for starting behavioral therapy.

### Intervention during the Late-phase treatment (Fig. 3)

**Fig. 3:**
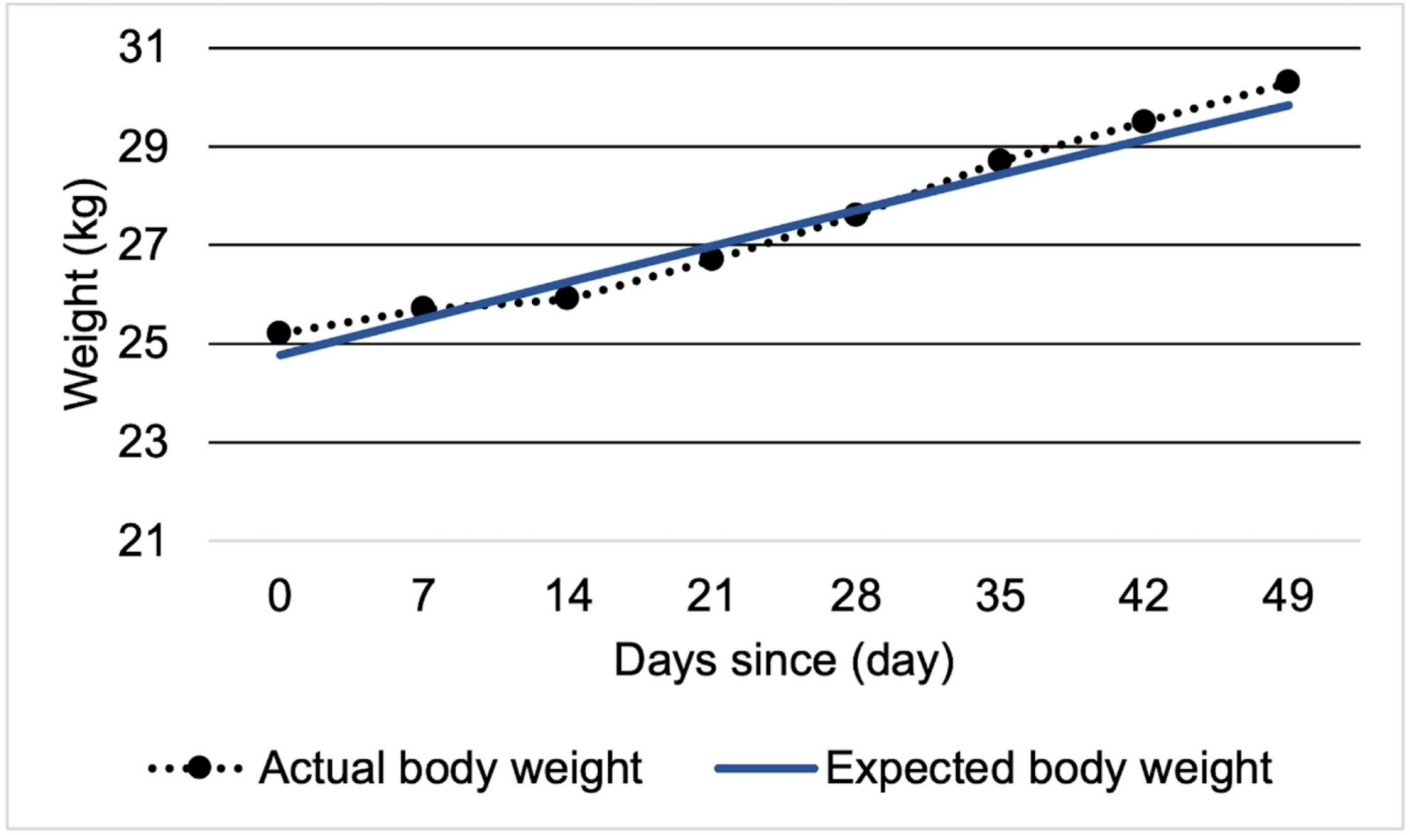
Weight trajectory after starting behavioral therapy. Body weight changes following the initiation of behavioral therapy. The axes are the same as in Fig. 2. Solid line: predicted weight (2000 kcal/day); dotted line: actual weight. Both lines are closely aligned

After the implementation of structured behavioral therapy, her weight began to increase, and the trajectory closely approximated the predicted curve (Fig. 3). Along with psychoeducation and nutritional counseling provided by a psychologist and dietitian, her nutrition was gradually transitioned from tube feeding to oral intake. The tube feeding ended on day 63. No subsequent weight loss was observed, and she maintained a weight above the inpatient treatment threshold defined by the Japanese Society for Eating Disorders [18] Finally, she was discharged at a body weight of 30 kg (BMI 14) on day 97.

## Discussion

The present case report illustrates the clinical application of a weight prediction model to support nutritional treatment in AN. In this case, a woman in her twenties with AN was readmitted with low body weight and liver dysfunction after diarrhea following the consumption of raw horse meat. In the initial phase, the patient insisted on continuing oral feeding and refused nasogastric nutrition despite the lack of weight gain. By introducing a weight prediction model based on the revised Harris-Benedict equation, both the patient and her family gained a better understanding of the treatment rationale. The model may help clarify the rationale for nasogastric feeding and behavioral therapy, supporting communication among the patient, her family, and the treatment team. This case may indicate the clinical utility of a weight prediction model in facilitating acceptance of nasogastric feeding and behavioral therapy in patients with AN. In real-world treatment settings, the mathematical model should be used in conjunction with established psychological interventions, rather than as a stand-alone approach.

This case report and model have four key strengths. First, it illustrates a rare example of using a calorie-based weight prediction equation to support communication about nutritional treatment in AN. Second, this model served as a practical tool that enabled the treatment team to visually and quantitatively explain the relationship between energy intake and weight change to the patient and her family. Third, its use promoted a shared understanding of the treatment plan and facilitated consensus regarding treatment during hospitalization among the patient, her family, and the treatment team. Fourth, this model is composed entirely of non-invasive parameters, such as current weight, height, age, sex, activity factor, stress factor, and caloric intake. These features differentiate this report and possibly warrant further prospective evaluation with calibration and external validation.

To date, no clinical application of a weight prediction model has been reported in patients with AN. Such a model may serve as a supportive tool for discussing nutritional strategies though its impact on clinical outcomes cannot be inferred from a single case. To our knowledge, no studies have utilized mathematical modeling for weight prediction; however, two prior investigations have explored prognostic factors in AN. One study was an observational investigation that identified prognostic factors for six-month BMI outcomes in SE-AN, reporting weight gain during hospitalization as significant predictors [11]. Another prospective study reported that the rate of weight gain during nutritional rehabilitation in AN was associated with treatment outcomes [12]. The present report describes only a single case, and the mathematical model was applied solely for the purpose of predicting weight gain; therefore, it was not possible to demonstrate any favorable impact on prognosis. However, because the mathematical model also allows the derivation of the rate of weight gain, it may provide a basis for adjusting the pace of nutritional supplementation to promote more favorable prognostic outcomes.

Although no prior studies have examined the use of mathematical models to facilitate treatment engagement in AN, previous reports have indicated that overly challenging and demanding treatment targets may overwhelm patients, thereby fostering resistance to treatment and resulting in counterproductive outcomes [4]. This highlights the importance of establishing attainable weight goals. In that study, the target weight was determined based on the clinician’s subjective judgment, which limited its objectivity and introduced considerable uncertainty. In contrast, the weight prediction model presented in this report may provide individualized and attainable treatment goals, thereby potentially enhancing patient motivation for treatment. However, the clinical application of this model requires careful consideration regarding the disclosure of numerical data. Qualitative research has suggested that weight exposure has a risk to foster preoccupation with numbers and increase compensatory behaviors [19, 20]. While the present case involved predicted future weight rather than current weight, the risk that disclosing numerical values itself could cause harm cannot be denied. Therefore, although this model may serve as a useful tool for motivation, it should not be implemented as a general procedure for all inpatients but rather reserved for selected cases based on careful clinical assessment.

Furthermore, no examples of non-invasive, dynamic, and quantitative nutritional indicators applicable to the clinical management of AN have been reported. Nutritional indicators such as albumin and prealbumin are commonly used [21] and in eating disorders, aspartate transaminase, alanine transaminase, cholesterol, and hemoglobin have also been considered useful markers [22]. However, these indicators are static and often require invasive blood sampling. Phase angle derived from bioimpedance analysis has been proposed as a metabolic marker [22], but it has not yet reached a level that allows for easy clinical application. In contrast, this prediction model relies solely on data that can be easily and promptly collected in clinical settings, thereby lowering the barrier to its implementation.

## Limitations

There are seven limitations for the present case report. First, this report describes a single patient with AN, and therefore the findings may not be generalizable to broader patient populations or clinical settings. Second, due to the observational and descriptive nature of the report, and the lack of a control condition, causal inferences regarding the effectiveness of the prediction model cannot be drawn. Third, several factors may compromise the model’s accuracy and reproducibility. Some parameters, such as clinician-estimated activity and stress factors, are subject to inter-rater variability [23]. In addition, discrepancies between predicted and actual weight may arise from inter-individual metabolic variability, and clinicians should explain both the possibility of such discrepancies and the approximate nature of the model’s predictions to patients in advance. Transient physical states of patients, such as dehydration or edema, can alter measured weight. This is particularly important in outpatient settings, where patients’ activity levels fluctuate significantly and require careful assessment. Additionally, the present model may have an aleatoric uncertainty because actual energy requirements vary substantially between individuals [16]. Fourth, the use of a prediction model might inadvertently trigger weight manipulation behaviors in some patients, introducing performance bias that may affect observed outcomes. Fifth, we did not assess changes in eating disorder psychopathology and quality of life. The present report focuses solely on short-term weight changes during inpatient treatment, and long-term follow-up data were not available. Therefore, no conclusions regarding treatment efficacy or broader clinical outcomes can be drawn. Sixth, in this case, evidence-based psychological therapies were not administered. Consequently, no conclusion can be drawn regarding whether the intervention based on this model offers benefits beyond those achieved with established psychotherapeutic approaches. Lastly, as this report highlights a successful case, the possibility of publication bias should be considered. Future studies with larger sample sizes and controlled designs are needed to validate the clinical use of calorie-based prediction models in AN.

## Conclusion

We reported a case in which a calorie-based weight prediction model supported communication about nutritional planning during inpatient care. The model facilitated understanding and agreement regarding treatment among the patient and her family. While the findings are limited to a single case and should be interpreted with caution, the model may serve as a valuable tool in clinical settings where objective assessment methods are limited.

## Supplementary Information

Below is the link to the electronic supplementary material.

Supplementary material 1.

## Data Availability

The datasets used during the current study are available from the corresponding author on reasonable request.

## Abbreviation

AN: Anorexia nervosa
BMI: Body mass index
