# Supplementary material for: Development of a calorie-based weight prediction equation for Anorexia nervosa: a case report
Source: J Eat Disord. 2026 Jan 9;14:44. doi: 10.1186/s40337-025-01520-7 (PMC12874902; doi:10.1186/s40337-025-01520-7)
Supplement: Supplementary file 1 — Supplementary material 1. [file 40337_2025_1520_MOESM1_ESM.docx]

**Supplementary Information**

Supplementary Equation

The Total energy expenditure(Et) is expressed as the product of Basal Energy Expenditure (BEE), activity factor (A), and stress factor (S).^1^

$$\begin{aligned} E_{t}=BEE\times A\times S\#\left( 1 \right) \end{aligned}$$

We assume S = 1 in the following calculations.

Let W(x) denote the body weight on day x, H the height (cm), and Y the age (years). Using the revised Harris-Benedict equation by Roza et al.,^2,3^ BEE is calculated as follows:

Male

$$BEE=88.362+13.397\times W\left( x \right)+4.799\times H-5.677\times Y$$

Female

$$BEE=447.593+9.247\times W\left( x \right)+3.098\times H-4.33\times Y$$

Assuming that approximately 7,000 kcal are required to gain 1 kg of body weight19, and that caloric intake per day is Ei, the following recurrence relation can be used to estimate daily weight change:

$$\begin{aligned} W\left( x+1 \right)=W\left( x \right)+\frac{E_{i}-E_{t}}{7000}\#\left( 2 \right) \end{aligned}$$

Where E_t_ is calculated from BEE as:

Male：$E_{t}=(88.3862+13.397\times W\left( x \right)+4.799\times H-5.677\times Y)\times A$

Female：$E_{t}=(447.593+9.247\times W\left( x \right)+3.098\times H-4.33\times Y)\times A$

Since the difference between E_i_ and E_t_ represents the surplus calories,

Male：$W\left( x+1 \right)=W\left( x \right)+\frac{E_{i}-(88.362+13.397\times W\left( x \right)+4.799\times H-5.677\times Y)\times A}{7000}$

Female：$W\left( x+1 \right)=W\left( x \right)+\frac{E_{i}-(447.593+9.247\times W\left( x \right)+3.098\times H-4.33\times Y)\times A)}{7000}$

Rewriting the recurrence relation, we obtain a difference equation of the form:

$$W\left( x+1 \right)=a\times W\left( x \right)+b$$

where a and b are constants dependent on height, age, caloric intake, and correction factors.

Thus, the general term for weight after n days, W(n), given daily caloric intake E_i_, is:

Male:

$$W\left( n \right)=\left( 1-\frac{13.397A}{7000} \right)^{n}W\left( 0 \right)+(\frac{E_{i}}{7000}-\frac{4.799HA}{7000}+\frac{5.677YA}{7000}-\frac{88.362A}{7000})\frac{{1-\left( 1-\frac{13.397A}{7000} \right)}^{n}}{1-\left( 1-\frac{13.397A}{7000} \right)}$$

Female:

$$W\left( n \right)=\left( 1-\frac{9.247A}{7000} \right)^{n}W\left( 0 \right)+(\frac{E_{i}}{7000}-\frac{3.098HA}{7000}+\frac{4.33YA}{7000}-\frac{447.593A}{7000})\frac{{1-\left( 1-\frac{9.247A}{7000} \right)}^{n}}{1-\left( 1-\frac{9.247A}{7000} \right)}$$

The source code implementing the calorie-based weight prediction model is publicly available on GitHub at <https://github.com/Riito9/weight-prediction> , versioned at commit ad74490 (linked at <https://github.com/Riito9/weight-prediction/tree/ad74490> and released as v1.0.1

A permanently archived version of this release is available via Zenodo <https://doi.org/10.5281/zenodo.16888704> (Version DOI: 10.5281/zenodo.16888704).

In addition, this case report was prepared in accordance with the CARE (CAse REports) guidelines.

**References**

1. Long, C. L., Schaffel, N., Geiger, J. W., Schiller, W. R. & Blakemore, W. S. Metabolic response to injury and illness: estimation of energy and protein needs from indirect calorimetry and nitrogen balance. *JPEN J. Parenter. Enteral Nutr.* **3**, 452–456 (1979).

2. Harris, J. A. & Benedict, F. G. A biometric study of human basal metabolism. *Proc. Natl. Acad. Sci. U. S. A.* **4**, 370–373 (1918).

3. Roza, A. M. & Shizgal, H. M. The Harris Benedict equation reevaluated: resting energy requirements and the body cell mass. *Am. J. Clin. Nutr.* **40**, 168–182 (1984).
